# Impact of ddPCR panel implementation on optimizing antimicrobial therapy management in critically ill patients with bloodstream infections

**DOI:** 10.3389/fcimb.2026.1850336

**Published:** 2026-07-06

**Authors:** Xiaoli Wang, Jing Wu, Chengcheng Shen, Rui Tian, Yunqi Dai, Jingjing Huang, De-en Zhong, Danting Fang, Jingyong Sun, Hongping Qu, Ruoming Tan

**Affiliations:** 1 Department of Critical Care Medicine, Ruijin Hospital, Shanghai Jiao Tong University School of Medicine, Shanghai, China; 2 Department of Pharmacy, Ruijin Hospital, Shanghai Jiao Tong University School of Medicine, Shanghai, China; 3 Department of Clinical Microbiology, Ruijin Hospital, Shanghai Jiao Tong University School of Medicine, Shanghai, China; 4 College of Health Science and Technology, Shanghai Jiao Tong University School of Medicine, Shanghai, China

**Keywords:** antimicrobial-stewardship, blood culture, bloodstream infection, digital droplet PCR (ddPCR), optimal antimicrobial therapy

## Abstract

**Background:**

Bloodstream infections (BSIs) represent a life-threatening complication in critically ill ICU patients. Rapid molecular diagnostic tools such as digital droplet PCR (ddPCR) may facilitate earlier targeted therapy, but their clinical impact on antimicrobial stewardship and patient outcomes remains inadequately characterized.

**Methods:**

This single-center, prospective cohort study enrolled ICU critically ill patients with confirmed BSIs between June 2021 and June 2024(ChiCTR2100047526). Patients were assigned to either a ddPCR-guided group, where antibiotic adjustments were made within 24 hours based on ddPCR results, or a standard-of-care group, where therapy was guided by conventional blood culture results. The primary outcome was 28−day mortality. Inverse probability of treatment weighting (IPTW) was used to adjust for potential confounding.

**Results:**

138 consecutive critically ill patients with BSIs were enrolled and assigned to either the ddPCR-guided group (n = 69) or the standard-of-care group (n = 69). DdPCR significantly reduced turnaround time compared to blood culture (median 0.3 days vs. 3.0 days; p < 0.001). Accordingly, the ddPCR-guided group achieved higher rates of optimal antibiotic therapy by 24 hours (94.2% vs. 46.4%; p < 0.001), antibiotic adjustments mainly involved vancomycin, caspofungin, and agents targeting multidrug-resistant organisms (MDROs). Although optimization rates equalized by day 5 and there was no significant difference in 28−day mortality between the two groups, ddPCR guidance was associated with significantly lower in-hospital mortality (36.2% vs. 55.1%; p = 0.026), particularly in patients with Gram-negative bacteremia (33.3% vs. 66.7%; p = 0.005), Gram-negative multidrug-resistant organism bacteremia (40.9% vs. 87.5%; p = 0.006), and persistent BSIs (34.2% vs. 72.0%; p = 0.003). After IPTW adjustment, standard-of-care management was associated with an increased in-hospital mortality rate compared with ddPCR-guided management (standard-of-care group vs. ddPCR-guided group: IRR = 1.46, 95% CI: 1.01–2.14, p = 0.045).

**Conclusions:**

Integrating ddPCR into routine ICU antimicrobial stewardship significantly shortened time to optimal therapy and improved survival, especially in high-risk BSI subgroups.

## Introduction

Bloodstream infections (BSIs) are a life-threatening condition with high prevalence, particularly in intensive care unit (ICU) settings (Holmes et al., 2025). In clinical practice, the argument is that diagnosing sepsis and BSIs can be challenging even for experienced clinicians. In septic shock cases, delayed and inadequate empiric antimicrobial treatment is associated with increased mortality (Liu et al., 2017; Seymour et al., 2017). Conventional methods for identifying and testing the susceptibility of microorganisms in blood cultures (BCs) typically require at least two days. During this period, patients may be administered antibiotics that are either ineffective or unnecessarily broad-spectrum (Zimmermann et al., 2024). It should be noted that, new molecular diagnostic tools have the potential to facilitate the earlier administration of targeted antimicrobial therapy, enable more timely de-escalation of broad-spectrum agents, and ultimately contribute to improved patient outcomes, especially in patients with septic shock (Holmes et al., 2025).

With the advent of novel diagnostic tools and therapeutic approaches, how to integrate these diagnostic tools into antibiotic stewardship measures remains a significant question that warrants further investigation (Alnimr, 2023). Our preliminary findings suggest that multiplex droplet digital PCR (ddPCR) could serve as a valuable adjunct to conventional blood culture, enabling rapid (2.5-hour) detection of BSIs and antimicrobial resistance (AMR) genes in ICU settings (Wu et al., 2022). Shen et al. demonstrated that integrating ddPCR-based pathogen load quantification with conventional biomarkers could optimize the clinical management of bloodstream infections. Their findings revealed two key advantages: (1) the combination of ddPCR with procalcitonin (PCT) levels significantly improved sepsis risk stratification, and (2) coupling ddPCR with interleukin-6 (IL-6) monitoring enhanced the predictive accuracy for disease progression trajectories (Yin et al., 2024). Despite its diagnostic potential, the clinical utility of ddPCR in bloodstream infection management remains inadequately characterized, with insufficient evidence regarding its impact on antimicrobial stewardship decisions and patient outcomes. Furthermore, clinical significance and optimal integration into clinical workflows have yet to be fully established.

To address this evidence gap, we conducted a prospective cohort study comparing antimicrobial utilization patterns and clinical outcomes in patients with BSIs managed with two distinct diagnostic approaches: (1) conventional culture with antimicrobial susceptibility testing (AST) versus (2) a rapid ddPCR-based panel detecting pathogens and selected resistance genes, followed by algorithm-guided antimicrobial therapy. Primary outcomes included: (i) 28−day mortality, (ii) appropriateness of antibiotic prescriptions, and (iii) subsequent therapeutic adjustments.

## Methods

### Study design

This single-center, pragmatic, prospective cohort study aims to evaluate the clinical utility of ddPCR testing in guiding appropriate antimicrobial therapy for BSIs in critically ill patients. The study was conducted in the general ICU of a tertiary academic medical center between June 8, 2021, and June 30, 2024, according to actual patient enrollment. This study was approved by the Ethics Committee of Ruijin Hospital, Shanghai Jiao Tong University School of Medicine (RJ2021-132), and all patients provided written informed consent before inclusion. This study was registered with the Chinese Clinical Trial Registry (ChiCTR2100047526).

### Population

This study prospectively enrolled consecutive patients admitted to the ICU who met the following inclusion criteria: (1) Age ≥18 years; (2) Suspected sepsis or BSIs; (3) Perform BC or ddPCR testing; (4) ICU length of stay exceeding 72 hours; (5) Definite BSIs with pathogens covered by the ddPCR panel; (6) In the ddPCR-guided group, patients with concurrent pathogen detection by both ddPCR and BC; and (7) In the standard-of-care group, BSIs were confirmed by BC alone.

The exclusion criteria comprised: (1) Pregnancy; (2) Moribund status; (3) Lack of informed consent; and (4) Identification of contaminants in blood cultures. To ensure data integrity, only the first eligible ICU admission and BSI event were considered during the study period. All enrolled patients underwent standardized blood sample collection procedures, and both ddPCR and BC testing were performed in accordance with previously established protocols and methods (Wu et al., 2022). The multiplex ddPCR test allows direct detection of the twelve most common bacterial pathogens or species, *Candida* species, and seven antimicrobial resistance (AMR) genes (including *bla*_KPC_, *bla*_NDM_, *bla*_IMP_, *bla*_OXA-48_, *mecA, vanA*, and *vanM*) from blood, at concentrations as low as 50 copies/mL (Pilot Gene Technologies. Hangzhou, China).

### Intervention and data collection

Targeted antibiotic therapy was adjusted within 24 hours of ddPCR result availability in the ddPCR-guided group, whereas in the standard-of-care group, definitive therapy adjustments were directed by treating clinicians until bloodstream culture results were available. Two independent intensivists assessed whether antibiotic therapy was optimized for each patient, with subsequent adjustments (escalation, de-escalation, or unchanged) guided by the ddPCR and BC results. Standardized clinical data were obtained from electronic medical records at enrollment and on day 5. Collected variables encompassed patient demographics, comorbidities, suspected infection site, organ dysfunction, surgical interventions, antibiotic regimens, and clinical outcomes. Furthermore, laboratory data were collected, including inflammatory markers (such as C-reactive protein, procalcitonin, and other relevant biomarkers), microbiological data (pathogens and their corresponding antimicrobial susceptibility results), and key outcome indicators.

### Definitions

Proven BSI in ddPCR-guided group required concordant positivity from both ddPCR and blood culture for the identical pathogen at the species level. Polymicrobial infection was defined as the detection of two or more distinct pathogenic microorganisms in the same sample (Nguyen et al., 2019). Persistent bacteremia or fungemia, defined by the persistence of positive blood cultures despite adequate antimicrobial therapy for ≥3 days (Mermel et al., 2009). Multidrug-resistant organisms (MDROs) include carbapenem-resistant *Klebsiella pneumoniae*, methicillin-resistant *Staphylococcus aureus*, and other bacteria resistant to at least one agent in at least three antimicrobial categories (Magiorakos et al., 2012).

Comorbidities were defined according to established literature, with malignancies including both hematologic malignancies and solid tumors. Immunosuppression was defined as antineoplastic therapy within 6 weeks, corticosteroids at a dose ≥20 mg prednisolone daily for at least 2 weeks or 30 mg prednisolone daily for at least 1 week before enrollment, and other immunosuppressants such as cyclophosphamide (Schweitzer et al., 2022). Optimal antimicrobial therapy was defined as administration of agents demonstrating both *in vitro* susceptibility (confirmed by antimicrobial susceptibility testing, interpreted by CLSI guidelines) and established clinical efficacy, while avoiding unnecessarily avoiding broad-spectrum regimens. The optimal treatment regimen was determined through independent evaluation and consensus by two intensivists (Nadjm et al., 2019). Antibiotic regimen modifications were categorized as follows:(1) escalation: replacement of narrow-spectrum agents with broader-spectrum alternatives, or the addition of supplemental antibiotics; (2) de-escalation: narrowing of the antimicrobial spectrum by switching to an agent with more targeted coverage, or by discontinuing redundant antibiotics; (3) unchanged: maintenance the original antibiotic protocol (Truong et al., 2022). The time to optimal antibiotic therapy was calculated in days and defined as the duration between study enrollment and the initiation of definitive antimicrobial treatment. For patients already receiving optimal therapy at enrollment, this interval was recorded as 0 days. Time to negativity for BC and ddPCR was calculated as the number of days from enrollment to the first documented negative result by either test.

### Outcomes

The primary outcome was 28-day mortality. Secondary outcomes were categorized as follows: (1) clinical outcomes, including in-hospital mortality; (2) process-of-care measures, including the proportion of patients on optimal antibiotic therapy at 24 hours and day 5, and the time to optimal therapy; and (3) microbiological outcomes, including time to blood culture negativity within 14 days and pathogen-stratified mortality analyses for Gram-negative bacteremia (and MDRO), Gram-positive bacteremia, fungemia, and persistent bacteremia.

### Sample size calculation

This prospective controlled trial compares the effect of ddPCR-guided versus standard-of-care antibiotic therapy on 28−day mortality in patients with BSIs. Based on preliminary data showing anticipated 28−day mortality rates of 15.0% in the ddPCR-guided group and 37.1% in the standard-of-care group according to the EUROBACT-2 international cohort study, we performed a sample size calculation (Tabah et al., 2023). Assuming a two-sided alpha of 0.05 and 80% power (β= 0.2), the formula for comparing two proportions indicated a need for 58 patients per group. Allowing for a 10% dropout rate, we increased enrollment to 69 patients per group, for a total of 138 patients. This calculation was further validated using G*Power (version 3.1), which confirmed the manual estimation.

### Statistical analysis

Data are expressed as median (IQR) for continuous variables and as frequency (%) for categorical variables. Groups were compared using the Wilcoxon rank-sum test for continuous variables and Chi-square test or Fisher’s exact test for categorical variables, as appropriate. To address potential selection bias, we employed inverse probability of treatment weighting (IPTW) based on propensity scores. Propensity scores—defined as the predicted probability of receiving ddPCR-guided therapy—were derived using multivariable logistic regression that included baseline covariates with a standardized mean difference (SMD) ≥0.1. Each patient was weighted by the inverse probability of their observed treatment to balance covariates between groups. Three Poisson regression models were subsequently fitted: a crude model; a model adjusting for variables with p ≤ 0.1 in univariate analysis; and a model with IPTW adjustment to control for confounding. All statistical analyses were conducted using R software (version 4.2.2). Two-sided p-values < 0.05 were considered statistically significant. Missing covariates were imputed using random forest imputation from the mice package in R (Van Buuren and Groothuis-Oudshoorn, 2011).

## Results

### Patient clinical characteristics

A total of 1024 patients with suspected BSIs were initially enrolled, of whom 210 had confirmed positive blood culture results. After applying the exclusion criteria, 138 patients were sequentially enrolled for final analysis. Based on clinical practice, these patients were divided into two groups: 69 in the ddPCR-guided group and 69 in the standard-of-care group. To balance baseline characteristics, an IPTW analysis was performed, yielding adjusted cohorts of 131.7 and 145.8 patients in the ddPCR-guided and standard-of-care groups, respectively (**Supplementary Figure 1**).The baseline demographic and clinical characteristics of the two groups before and after IPTW are summarized in **Table 1**. In the unadjusted cohorts, approximately 50% of BSIs originated from the abdominal infections, followed by respiratory tract infections. However, the ddPCR-guided group had more Gram-negative infections (58.0% vs. 43.5%; SMD = 0.293), while the standard-of-care group had more Gram-positive infections (39.1% vs. 23.2%; SMD = 0.349). White blood cell (WBC) count was significantly lower in the ddPCR-guided group (9.1 vs. 11.3; P = 0.041; SMD = 0.311). Other indicators were well balanced (SMD < 0.2). After IPTW adjustment, all covariates were well balanced between the two groups. The initial imbalances in pathogen distribution and WBC count were resolved. All other demographic and clinical variables exhibited SMDs well below 0.2 (most < 0.1), indicating excellent covariate balance. Thus, IPTW effectively minimized selection bias, allowing for robust outcome assessment.

**Table 1:** Baseline characteristics of the unadjusted and IPTW-adjusted populations.

	Unadjusted				IPTW-adjusted			
Characteristics	ddPCR-guided group (n=69)	Standard-of-care group(n=69)	P value	SMD	ddPCR-guided group (n=131.7)	Standard-of-care group(n=145.8)	P value	SMD
Age (years), median (IQR)	73 (64,81)	71 (58,82)	0.590	0.123	72.0 (60.0,80.2)	68.0 (57.0,82.0)	0.924	0.024
Male, n (%)	46 (66.7)	41 (59.4)	0.481	0.151	90.8 (68.9)	100.7 (69.0)	0.989	0.003
Comorbidities								
Hypertension, n (%)	39 (56.5)	41(59.4)	0.863	0.059	81.0 (61.5)	85.7 (58.8)	0.813	0.055
Diabetes mellitus, n (%)	20 (29.0)	18 (26.1)	0.849	0.065	35.0 (26.6)	31.6 (21.7)	0.549	0.116
Coronary heart disease, n (%)	11 (15.9)	16 (23.2)	0.391	0.183	29.9 (22.7)	22.7 (15.6)	0.451	0.182
CKD, n (%)	6 (8.7)	9 (13.0)	0.584	0.140	10.7 (8.1)	12.7 (8.7)	0.902	0.021
Malignant tumor, n (%)	35 (50.7)	26 (37.7)	0.170	0.265	55.9 (42.5)	60.6 (41.5)	0.934	0.019
COPD, n (%)	1 (1.4)	2 (2.9)	1.000	0.100	1.0 (0.8)	2.0 (1.4)	0.629	0.060
Immunosuppressive, n (%)	10 (14.5)	10 (14.5)	1.000	<0.001	19.6 (14.9)	16.0 (10.9)	0.516	0.117
Source of targeted organisms*			0.373	0.402			0.519	0.305
Abdominal, n (%)	38 (55.1)	34 (49.3)			68.0 (51.6)	90.0 (61.7)		
Respiratory, n (%)	24 (34.8)	32 (46.4)			55.8 (42.3)	51.8 (35.5)		
Urine, n (%)	2 (2.9)	0.0 (0.0)			2.0 (1.5)	0.0 (0.0)		
Skin and soft, n (%)	1(1.4)	0.0 (0.0)			1.0 (0.8)	0.0 (0.0)		
Catheter related, n (%)	3 (4.3)	3 (4.3)			4.0 (3.0)	4.0 (2.8)		
Others, n (%)	1 (1.4)	0.0 (0.0)			1.0 (0.8)	0.0 (0.0)		
Severity of illness								
Mechanical ventilation, n (%)	54 (78.3)	56 (81.2)	0.832	0.072	103.0 (78.2)	115.2 (79.0)	0.934	0.019
Septic shock, n (%)	53 (76.8)	50 (72.5)	0.696	0.100	100.8 (76.5)	106.2 (72.8)	0.709	0.084
Surgery performed before 30 days of inclusion, n (%)	46 (66.7)	37 (53.6)	0.164	0.269	76.0 (57.7)	96.1 (65.9)	0.453	0.169
SOFA score, median (IQR)	9 (6,12)	9 (5,14)	0.528	0.134	9 (6,13)	6 (5,13)	0.312	0.124
APACHE II score, median (IQR)	19 (15,25)	19 (15,30)	0.720	0.102	21 (15,25)	18 (14,26)	0.305	0.135
Identified pathogen**								
Gram-negative organism, n (%)	40 (58.0)	30 (43.5)	0.125	0.293	65.9 (50.0)	82.9 (56.9)	0.546	0.137
Gram-positive organism, n (%)	16 (23.2)	27(39.1)	0.066	0.349	39.3 (29.9)	36.8 (25.2)	0.654	0.104
Fungus, n (%)	13 (18.8)	16 (23.2)	0.676	0.107	26.5 (20.1)	30.1 (20.7)	0.946	0.014
Clinical indicators								
C Reactive Protein, mg/L, median (IQR)	106 (46,205)	92 (51,141)	0.218	0.313	83 (39,153)	93 (51,154)	0.764	0.042
White Blood Cell,10^9/L, median (IQR)	9.1 (5.7,14.0)	11.3 (7.3,15.6)	0.041*	0.311	10.1 (6.1,15.3)	10.5 (5.1,14.0)	0.535	0.179
Lymphocyte, 10^9/L, median (IQR)	0.6 (0.4,0.9)	0.6 (0.4,1.0)	0.792	0.098	0.7 (0.4,1.2)	0.5 (0.3,0.8)	0.098	0.068
Platelet, 10^9/L, median (IQR)	129 (77,224)	120 (59,221)	0.878	0.023	144 (77,223)	114 (62,213)	0.638	0.038
Procalcitonin, ng/mL, median (IQR)	2.2 (0.5,11.0)	1.6 (0.5,6.0)	0.485	0.043	1.6 (0.4,8.0)	1.1 (0.4,4.6)	0.510	0.057
Pro-BNP, ng/mL, median (IQR)	2067 (708,5377)	2358 (738,9587)	0.494	0.221	2941(711,5186)	1316 (529, 5258)	0.295	0.026
Pre-albumin, g/L, median (IQR)	84 (61,126)	101 (79,131)	0.084	0.152	97 (61,157)	87 (73,121)	0.894	0.271
Creatinine, μmol/L, median (IQR)	86 (58,112)	84 (60,163)	0.547	0.122	97 (58,110)	76 (61,137)	0.948	0.011
Total Bilirubin, μmol/L, median (IQR)	20 (14,47)	26 (14,46)	0.705	0.129	23 (16,44)	20 (16,35)	0.536	0.011
Fibrinogen, g/L, median (IQR)	3.4 (2.4,4.7)	3.6 (2.4,5.1)	0.759	0.045	3.0 (2.4,4.4)	4.0 (2.6,5.7)	0.129	0.167
D-dimer, μg/L, median (IQR)	4.4 (2.4,6.6)	4.4 (2.4,8.8)	0.393	0.169	4.6 (2.5,8.1)	4.2 (2.4,7.3)	0.794	0.059

IPTW, inverse probability of treatment weighting; SMD, standardized mean difference; IQR, interquartile range; CKD, chronic kidney disease; COPD, chronic obstructive pulmonary disease; SOFA, sequential organ failure assessment; APACHE II, acute physiology and chronic health evaluation II; BSI, bloodstream infection. Values are presented as the median (IQR), or number of subjects (percentage of the column total).

*In the ddPCR-guided group, patient infection characteristics were as follows: one patient had concurrent BSI originating from the respiratory tract, skin/soft tissue, and a catheter-related source; another patient's BSI was associated with both respiratory and catheter-related sources; and a third patient’s BSI was of abdominal and respiratory origin. In the standard-of-care group, one patient presented with BSI involving respiratory, skin/soft tissue, and catheter-related sources, while another patient's BSI linked to both respiratory and central nervous system (CNS) infection.

**In the ddPCR-guided group, two patients developed BSIs caused by *Acinetobacter baumannii* and *Klebsiella pneumoniae*. In the standard-of-care group, one patient had a BSI involving *Enterococcus* species and *Acinetobacter baumannii*; two patients were co-infected with *Enterococcus* species and *Pseudomonas aeruginosa*; and one patient presented with a BSI caused by *Acinetobacter baumannii* and *Staphylococcus aureus.*

### Pathogen distribution and resistance genes

A total of 144 pathogen isolates were detected among the 138 enrolled patients, with polymicrobial infections identified in 4.3% (6/138) of cases (2 in the ddPCR-guided group and 4 in the standard-of-care group). The most prevalent pathogens were *Candida* spp., *Enterococcus* spp., and *Klebsiella pneumoniae*, each accounting for approximately 20% of infections (**Table 2**). The ddPCR-guided group had a higher proportion of Gram-negative organisms, including *K. pneumoniae* (23.9% vs. 13.7%) and *S. maltophilia* (14.1% vs. 5.5%), whereas the standard-of-care group had a higher proportion of *Enterococcus* (26.0% vs. 15.5%). Candida species prevalence was similar between groups (18.3% vs. 21.9%).

**Table 2:** Summary of pathogens and AMR genes detected in different groups.

Pathogens	All patients (n=138)	ddPCR-guided group (n=69)	Standard-of-care group(n=69)
*Any pathogens*	144	71	73
Single pathogen*, n (%)	132(91.7)	67(94.4)	65(89.0)
*Klebsiella pneumoniae*	27(18.8)	17(23.9)	10(13.7)
*Acinetobacter baumannii*	14(9.7)	6(8.5)	8(11.0)
*Pseudomonas aeruginosa*	3(2.1)	2(2.8)	1(1.4)
*Escherichia coli*	4(2.8)	2(2.8)	2(2.7)
*Proteus mirabilis*	1(0.7)	1(1.4)	0
*Stenotrophomonas maltophilia*	14(9.7)	10(14.1)	4(5.5)
*Serratia marcescens*	1(0.7)	0	1(1.4)
*Enterococcus*	30(20.8)	11(15.5)	19(26.0)
*Staphylococcus aureus*	6(4.2)	4(5.6)	2(2.7)
*Coagulase-negative Staphylococcus*	3(2.1)	1(1.4)	2(2.7)
*Candida*	29(20.1)	13(18.3)	16(21.9)
Multidrug-resistant bacteria, n (%)	44(30.6)	26(36.6)	18(24.7)
Multiple pathogens, n (%)	6	2	4
*Klebsiella pneumoniae+ Acinetobacter baumannii*	2(33.3)	2(100.0)	0
*Acinetobacter baumannii + Enterococcus*	1(16.7)	0	1(25.0)
*Pseudomonas Aeruginosa+ Enterococcus*	2(33.3)	0	2(50.0)
*Acinetobacter baumannii + Staphylococcus aureus*	1(16.7)	0	1(25.0)
*Bla_KPC_ + Klebsiella Pneumoniae*	8	8	/
*Bla_NDM_/Bla_IMP_ + Klebsiella Pneumoniae*	2	2	/
*mecA*	4	4	/
*mecA+Staphylococcus aureus*	3	3	/
*mecA+Coagulase-negative Staphylococcus*	1	1	/

All data presented as n (%). AMR, antimicrobial resistance.

Regarding antimicrobial resistance, the ddPCR-guided group had a higher rate of MDR bacteria compared to the standard-of-care group (36.6% vs. 24.7%). Beyond pathogen identification, ddPCR detected 14 AMR gene-positive episodes concordant with conventional blood culture results. Among these, 8 samples were positive for *bla*_KPC_ in *K. pneumoniae*, 2 for *bla*_NDM_/*bla*_IMP_ in *K. pneumoniae*, and 4 carried the *mecA* gene (3 in *S. aureus* and 1 in coagulase-negative *Staphylococci*) (**Table 2**).

### Antibiotic adjustments, optimal therapy rates at 24h and day 5

**Figure 1:**
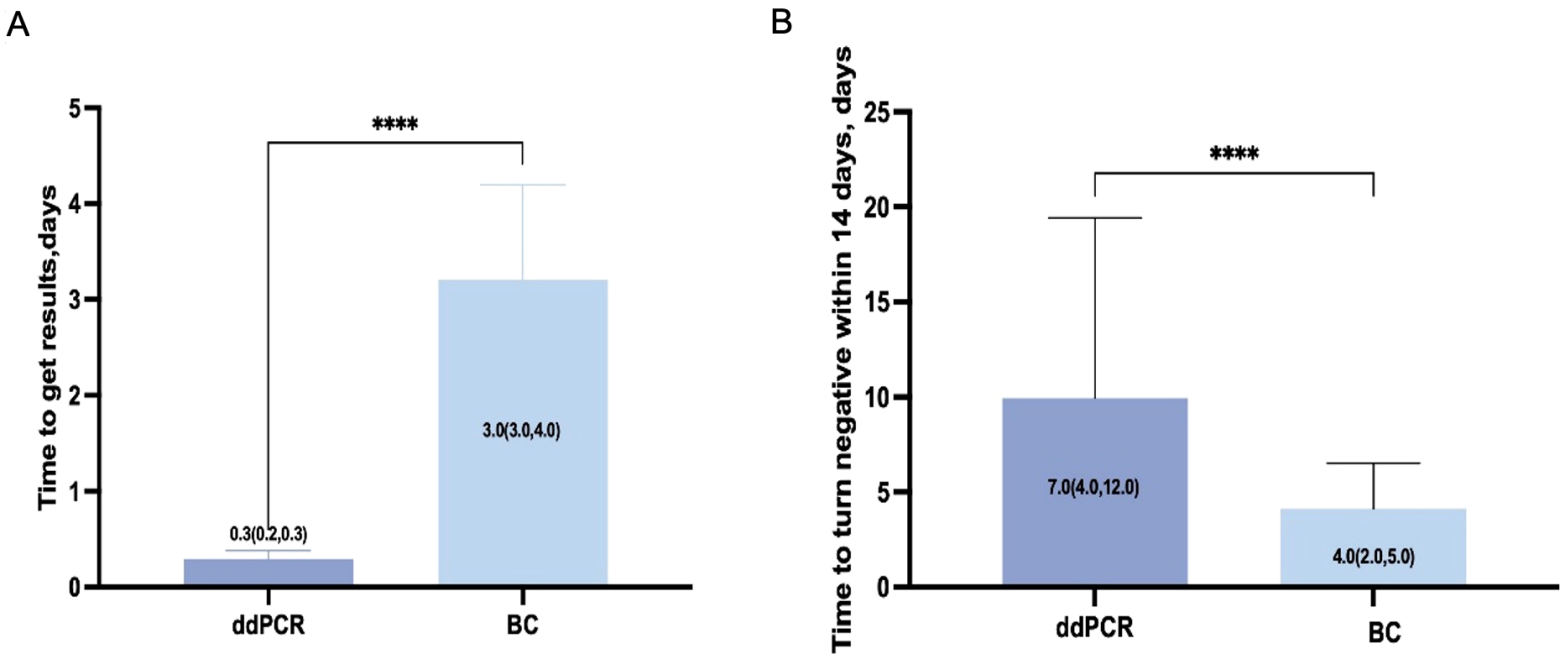
The turnaround time for pathogen detection and the time to negative conversion within 14 days were compared between ddPCR and blood-culture methods in the ddPCR-guided group. **(A)**. Turnaround time (TAT) for pathogen detection: ddPCR versus blood culture, and **(B)**. Time to negative conversion within 14 days: ddPCR versus blood culture. ****p < 0.0001.

As shown in **Figure 1A**, ddPCR had a significantly shorter median turnaround time than blood culture (0.3 days [IQR: 0.2–0.3] vs. 3.0 days [IQR: 3.0–4.0]; p < 0.001), facilitating earlier clinical decision-making. **Figure 1B** presents the time to negative conversion within 14 days, which was significantly longer in the ddPCR-guided group compared to the blood culture-guided group (median 7.0 days [IQR: 4.0–12.0] vs. 4.0 days [IQR: 2.0–5.0]; p < 0.001).

**Figure 2:**
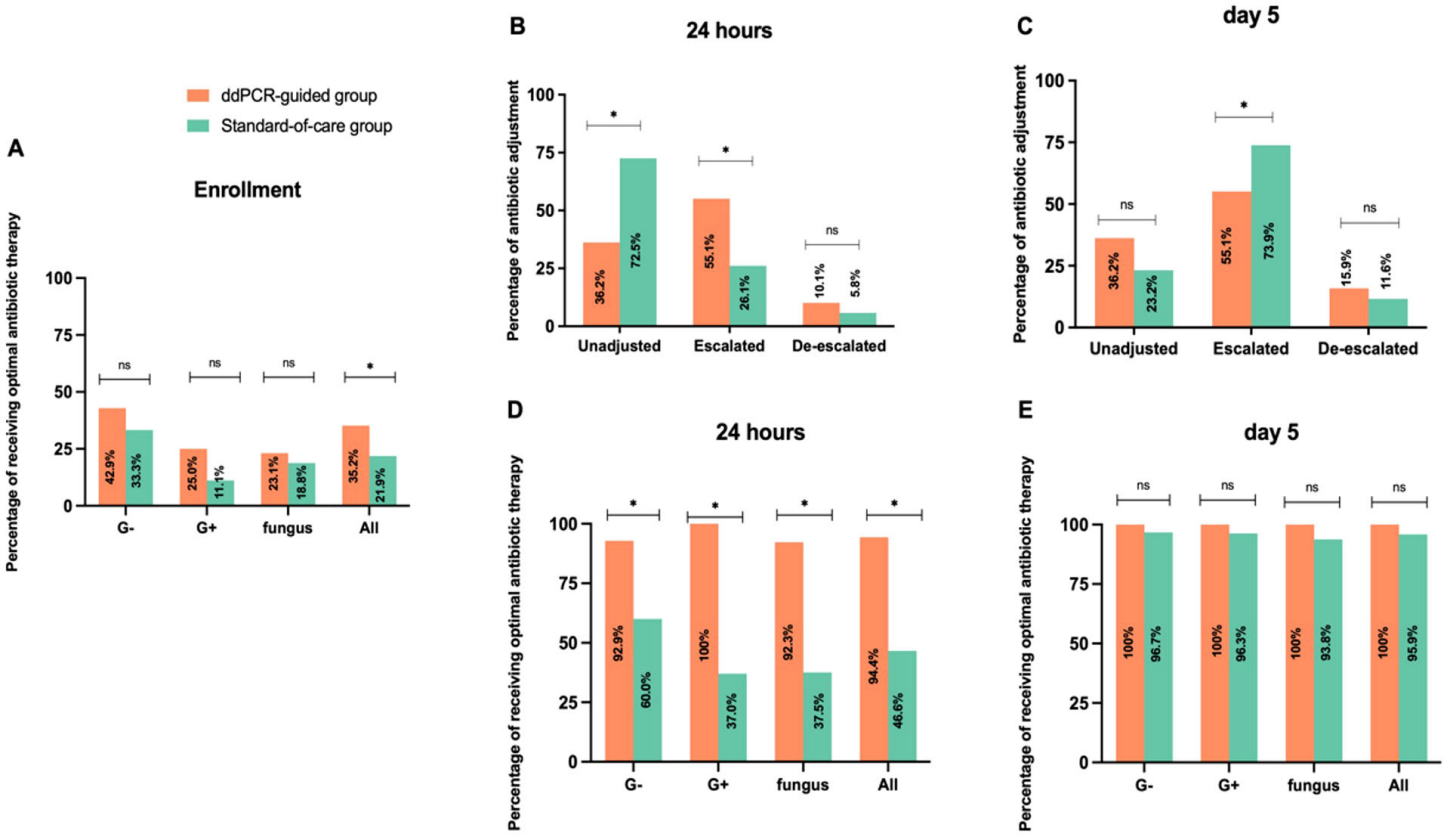
Differences between the ddPCR-guided and standard-of-care groups were assessed based on antibiotic adjustments and the cumulative proportion of patients receiving optimal therapy, evaluated at enrollment, 24 hours, and day 5. **(A)**. Cumulative proportion of patients achieving optimal antibiotic therapy on enrollment^1^. **(B)**. Antibiotic adjustments on 24 hours between the two groups. **(C)**. Antibiotic adjustments on day 5 between the two groups^2^. **(D)**. Cumulative proportion of patients achieving optimal antibiotic therapy on 24 hours. **(E)**. Cumulative proportion of patients achieving optimal antibiotic therapy on day 53. Notes: G: Gram-negative pathogens; G^*^: Gram-positive pathogens. 1. The ddPCR-guided group evaluated 42 Gram-negative, 16 Gram-positive, and 13 fungal pathogens, whereas the standard-of-care group evaluated 30 Gram-negative, 27 Gram-positive, and 16 fungal pathogens. 2. In the ddPCR-guided group, five patients received simultaneous escalation and de-escalation of antimicrobial therapy. In the standard-of-care group, five patients experienced concurrent escalation and de-escalation. Additionally, one patient underwent two separate de-escalation events, another had two separate escalation events, and one patient presented with a co-infection involving two pathogens: one was adequately covered by the initial treatment upon admission, while the other necessitated escalation of antimicrobial therapy. 3. **Figures 2A, D, E** calculated according to the number of pathogens. **Figures 2B, C** according to the number of patients. * p < 0.05; ns: not significant.

At enrollment (**Figure 2A**), the ddPCR-guided group had a higher baseline rate of optimal antibiotic therapy compared to the standard-of-care group, both overall (35.2% vs. 21.9%; p< 0.05) and across all pathogen categories. Within 24 hours of enrollment (**Figure 2B**), the ddPCR-guided group demonstrated more frequent antibiotic adjustments. Escalation therapy occurred in 55.1% (38/69) of ddPCR-guided patients versus 26.1% (18/69) in the standard-of-care group (p < 0.05). Unchanged therapy was more common in the standard-of-care group (72.5% vs. 36.2%; p < 0.05). De-escalation was infrequent in both groups during this early period. By day 5 (**Figure 2C**), the standard-of-care group showed a substantial increase in therapy escalation (73.9%, 51/69), surpassing the ddPCR-guided group (55.1%, 38/69). De-escalation rates remained low in both groups but were slightly higher in the standard-of-care group (15.9% vs. 11.6%). At 24 hours (**Figure 2D**), the ddPCR-guided group achieved a significantly higher rate of optimal therapy by pathogen type (94.4% [67/71] vs. 46.6% [34/73]; p < 0.001), a benefit observed across all pathogen categories. By day 5, however, this difference had equalized, with both groups achieving comparable rates of optimal therapy overall and by pathogen type, reflecting the delay clinical decision-making in the conventional approach.

### Sankey diagram and antibiotic adjustments

**Figure 3:**
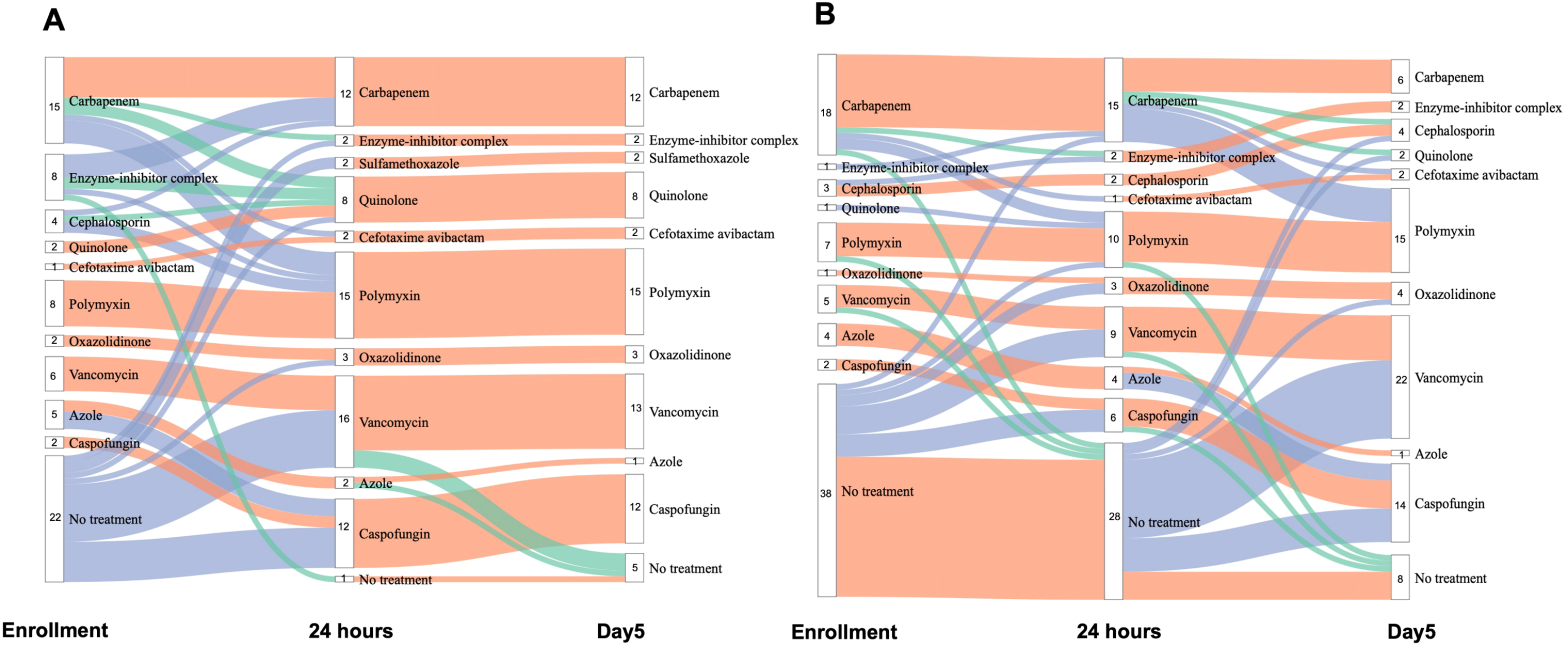
Flow diagram of antibiotic regimen adjustments in the ddPCR-guided **(A)** and standard-of-care **(B)** groups. Flows originate from pre-enrollment regimens (left), proceed to 24h prescriptions (middle), and terminate on day-5 (right) in optimal regimens guided by ddPCR or blood culture . Purple indicates escalation, orange represents unadjusted, and green denotes de-escalation.

**Figure 3** illustrates the path from empirical to optimized antibiotic therapy using Sankey diagrams. In the ddPCR-guided group, treatment optimization was rapid: 55.1% (38/69) of patients received antibiotic escalation within 24 hours of ddPCR results. These adjustments were predominantly targeted therapies, including vancomycin for Gram-positive pathogens (26.3% [10/38]), caspofungin for fungi (26.3% [10/38]), and MDRO-directed regimens (21.1% [8/38]). De-escalation occurred in 11 patients over five days, mainly involving the discontinuation of carbapenems or β-lactam/β-lactamase inhibitor combinations (54.5%) (**Figure 3A**). Among patients with *bla*_KPC_-positive *K. pneumoniae*, 2 had antibiotic escalation based on test results while the other 6 had adequate coverage and continued treatment; among those with *bla*_NDM_/*bla*_IMP_-positive *K. pneumoniae*, 1 was escalated from a third-generation cephalosporin to colistin and the other was already covered; and among patients with *mecA*-positive *S. aureus* or coagulase-negative *Staphylococci*, 1 was escalated to vancomycin while the rest had initial coverage.

In the standard-of-care group, empirical antibiotic therapy failed to cover 38 patients (55.1% [38/69]) at enrollment. Within 24 hours, 18 patients (26.1%) required targeted escalation, most commonly with vancomycin (26.3% [5/19]), followed by polymyxins and caspofungin (each 21.1% [4/19]). By day 5, escalation was required in 73.9% (51/69) of patients, with vancomycin (36.5% [19/52]), caspofungin (25.0% [13/52]), and MDRO-targeted therapy (23.1% [12/52]) being the most frequent. Among the 9 de-escalation events observed, carbapenem discontinuation accounted for about half of cases (44.4% [4/9]) (**Figure 3B**).

Notably, for initially uncovered pathogens such as Gram-positive bacteria, MDROs, and fungi, the ddPCR-guided approach allowed clinicians to initiate optimal antimicrobial therapy by 24 hours, while the standard-of-care group did not complete these adjustments until day 5.

### Clinical outcomes

Although no significant difference in 28−day mortality was observed between the two groups, the ddPCR-guided group exhibited a significantly lower in-hospital mortality rate compared to the standard-of-care group in **Table 3** (36.2% vs. 55.1%, p = 0.026). Overall, the ddPCR-guided group achieved the optimal antibiotic therapy significantly faster than the standard-of-care group (median 1 day vs. 2 days; p < 0.001). Pathogen-specific analysis revealed a marked mortality benefit in the ddPCR-guided group for patients with Gram-negative infections (33.3% vs. 66.7%; p = 0.005) and, notably, for those with Gram-negative MDROs (40.9% vs. 87.5%; p = 0.006). In contrast, no significant differences in mortality were observed for Gram-positive or fungal infections. Furthermore, the ddPCR-guided approach was associated with a substantially reduced mortality rate in patients with persistent bloodstream infections (34.2% vs. 72.0%; p = 0.003).

**Table 3:** Primary and secondary outcomes.

Outcomes	ddPCR-guided group (n=69)	Standard-of-care group (n=69)	P value
Primary outcome			
28-day mortality of all patients, n (%)	15(21.7)	22(31.9)	0.179
Secondary outcomes			
In-hospital mortality of all patients, n (%)	25(36.2)	38(55.1)	0.026*
Receive optimal antibiotic therapy within 24 hours, n (%)^1^	65(94.2)	32(46.4)	<0.001*
Receive optimal antibiotic therapy on day 5, n (%)	69(100)	66(95.7)	0.243
Time to optimal antibiotic therapy, days, median (IQR)	1(0,1)	2(1,4)	<0.001*
BC turned negative within 14 days, n (%)	54(78.3)	59(85.5)	0.377
In-hospital mortality of gram-negative bacteria infected patients, n (%)^3,8^	14(33.3)	20(66.7)	0.005*
In-hospital mortality of gram-negative multidrug-resistant bacteria infected patients, n (%)^4^	9(40.9)	14(87.5)	0.006*
In-hospital mortality of gram-positive bacteria infected patients, n (%)^5^	6(37.5)	13(48.1)	0.497
In-hospital mortality of fungi infected patients, n (%)^6^	5(38.5)	9(56.3)	0.462
In-hospital mortality of persistent BSI patients, n (%)^7^	13(34.2)	18(72.0)	0.003*

Values are presented as the median (IQR), or number of subjects (percentage of the column total). BSI,bloodstream infection.

^1^ Antibiotic therapy was considered optimal if it was not only active but also not excessively broad-spectrum.

^2^ Assessed in 54 patients in ddPCR-guided group and 59 patients in standard-of-care group, while 15 patients in ddPCR-guided group and 10 patients in standard-of-care group didn’t turn negative at the time of discharge or death or within 14 days after enrollment.

^3^ Assessed 42 gram-negative bacteria infected patients in ddPCR-guided group and 30 patients in standard-of-care group.

^4^ Assessed 22 gram-negative multidrug-resistant bacteria infected patients in ddPCR-guided group, 16 patients in standard-of-care group.

^5^ Assessed 16 gram-positive bacteria infected patients in ddPCR-guided group and 27 patients in standard-of-care group; Patients with polymicrobial infections were counted as gram-negative bacteria.

^6^ Assessed 13 fungi bacteria infected patients in ddPCR-guided group and 16 patients in standard-of-care group.

^7^ Assessed 38 persistent BSI patients in ddPCR-guided group,25 patients in standard-of-care group.

^8^ In standard-of-care group,4 patients co-infected with gram-negative and positive bacteria died.

A patient-specific propensity score was derived using multivariable logistic regression to predict the probability of assignment to the ddPCR-guided group, incorporating major confounding variables detailed in **Supplementary Table 1** and **Supplementary Figure 2**. Three sequential Poisson regression models were constructed. In the unadjusted model (Model 1), no significant difference in mortality was observed between the two groups. After adjusting for variables with p ≤0.1 in univariable Poisson regression (Model 2), the incidence rate ratio (IRR) remained numerically elevated but did not reach statistical significance (IRR = 1.59, 95% CI: 0.95–2.72, p = 0.082). However, in the IPTW balanced population in Model 3, standard-of-care management was significantly associated with a higher in-hospital mortality rate compared with ddPCR-guided management (standard-of-care group vs. ddPCR-guided group: IRR = 1.46, 95% CI: 1.01–2.14, p = 0.045), indicating that ddPCR guidance was significantly associated with a reduced risk of in-hospital mortality (**Table 4**).

**Table 4:** Results of Poisson regression models between the ddPCR-guided group and standard-of-care group.

Model	IRR (95% CI)	p-value
Model 1	1.52(0.92, 2.55)	0.10
Model 2	1.59(0.95, 2.72)	0.082
Model 3	1.46(1.01, 2.14)	0.045

IRR, Incidence Rate Ratio; CI, Confidence Interval.

Model 1 was unadjusted. Model 2 was adjusted for the use of mechanical ventilation, septic shock, platelet count, pro-B-type natriuretic peptide (pro-BNP), prealbumin, total bilirubin, and serum creatinine based on Model 1. Model 3 further used inverse probability of treatment weighting (IPTW) to balance covariates, with weight calculated based on the following variables according to standardized mean difference (SMD) in baseline between groups: gender, age; history of coronary heart disease (CHD), chronic obstructive pulmonary disease (COPD), chronic kidney disease (CKD) and malignant tumor; main source of targeted organisms, Gram-positive organism, Gram-negative organism, fungal organism, optimal antibiotic therapy on enrollment, septic shock, APACHE II score, SOFA score, surgery performed before 30 days of inclusion, C-reactive protein (CRP), white blood cell count (WBC), pro-BNP, prealbumin, total bilirubin, serum creatinine, and D-dimer.

Additionally, we serially measured key systemic inflammatory markers following the 5-day treatment course in both groups. The ddPCR-guided group exhibited a significantly greater reduction in C-reactive protein (CRP) from baseline to day 5 compared to the standard-of-care group (p = 0.009). No other biomarkers showed significant intergroup differences in their dynamic changes (**Supplementary Table 2**).

## Discussion

In our study, ddPCR significantly reduced the turnaround time for pathogen detection compared to conventional methods (0.3 days vs. 3.0 days; p < 0.001), enabling earlier initiation of targeted therapy. Notably, we observed that ddPCR maintained positivity longer than blood culture (median 7.0 days vs. 4.0 days; p < 0.001), indicating the need for further research to establish its role in treatment monitoring and guiding antibiotic discontinuation. The ddPCR-guided group achieved earlier antimicrobial regimen adjustments, primarily targeting Gram-positive pathogens, MDROs and fungi, resulting in significantly higher antibiotic optimization rates by 24 hours (94.2% vs. 46.4%; p < 0.001). Although optimization rates equalized by day 5 and there was no significant difference in 28−day mortality between the two groups, ddPCR guidance was associated with significantly lower in-hospital mortality, particularly in high-risk subgroups. Mortality was markedly reduced in patients with Gram-negative bacteremia (33.3% vs. 66.7%; p = 0.005), Gram-negative MDRO bacteremia (40.9% vs. 87.5%; p = 0.006), and persistent bloodstream infections (34.2% vs. 72.0%; p = 0.003). After adjusting for confounding via IPTW, the ddPCR-guided strategy was significantly associated with a reduced risk of in-hospital mortality (IRR = 1.46, 95% CI: 1.01–2.14, p = 0.045).

Among critically ill patients, BSIs may progress to sepsis and potentially life-threatening septic shock. The current Surviving Sepsis Campaign (SSC) guidelines recommend initiating antibiotic therapy within one hour for patients with septic shock or those exhibiting a high likelihood of sepsis (Evans et al., 2021). Several studies suggest using combination therapy in empiric treatment for suspected or confirmed septic shock in ICU patients to enhance pathogen coverage (Torisson et al., 2021; Tanha et al., 2024; Gupta et al., 2026). However, approximately 30-35% of patients treated empirically for sepsis are subsequently found to have either nonbacterial infections or non-infectious conditions that present with sepsis-like symptoms (Shappell et al., 2021). This substantial rate of misdiagnosis contributes to inappropriate antibiotic use, which in turn drives the emergence and spread of antimicrobial resistance. In another clinical scenario, culture-negative status was paradoxically associated with increased mortality in septic shock patients (Chang et al., 2024; Zizzo et al., 2025). The absence of pathogen identification and antimicrobial susceptibility data compromised multiple aspects of clinical management: (1) delayed appropriate antibiotic therapy initiation, especially in MDRO infections, (2) challenges in monitoring treatment response, and (3) difficulties in guiding antibiotic de-escalation or discontinuation decisions (Alnimr, 2023; Maraolo et al., 2025). Notably, many existing studies on bloodstream infections systematically exclude immunocompromised patients, persistent bacteremia, polymicrobial infections, and drug-resistant pathogens (Papp et al., 2023; Lee et al., 2025; The BALANCE Investigators, 2025). These high-risk ICU populations warrant particular attention as they are more prone to develop severe complications and poor outcomes. All findings suggest that, rapid, accurate, and affordable rapid molecular diagnostic technologies such as ddPCR should be implemented as complementary measures to conventional testing in critically ill patients with septic shock, enabling prompt diagnosis and guiding the antimicrobial stewardship workflow (Zimmermann et al., 2024; Alcolea-Medina et al., 2025). Subsequent culture results can then guide regimen optimization through either streamlining or de-escalation of the initial antibiotic protocol.

Studies evaluating the clinical impact of rapid molecular diagnostic techniques have been limited by their observational study designs and reliance on historical controls. While these studies suggest that antimicrobial use, length of stay, mortality, and/or cost may be reduced through the use of rapid diagnostic tests (Peri et al., 2024; Singla et al., 2025). Combining antimicrobial stewardship interventions with metagenomic next-generation sequencing and MALDI-TOF shortened the time to definitive therapy, accelerated initial antibiotic adjustments, and was associated with reduced post-culture hospitalization duration (Nadjm et al., 2019; Xu et al., 2024). Respiratory metagenomics for ICU patients showed good performance and rapid turnaround time, resulting in antimicrobial therapy changes in 28% (30/107) of samples (Alcolea-Medina et al., 2025). In a recent study on critically ill immunocompromised patients with pneumonia, the multiplex PCR (mPCR) assay demonstrated notable diagnostic and clinical utility. Specifically, mPCR facilitated modifications to antibiotic therapy in 17.5% of cases, predominantly through de-escalation, thereby optimizing antimicrobial stewardship (Contier et al., 2025).

To date, there have been very few clinical trials evaluating ddPCR-guided therapy and its impact on antibiotic stewardship and patient outcomes in BSIs. A daily re-evaluation of the effectiveness of the antibiotic regimen based on the available information is an important strategy during BSI treatment, especially during the first 24 h of treatment (Niwa et al., 2016). By embedding ddPCR within an antimicrobial-stewardship framework, targeted therapy was initiated roughly 2.5 days earlier than with blood-culture-based diagnostics, and 94.2% of patients were receiving an appropriate regimen by 24 hours. Notably, ddPCR enabled significantly earlier initiation of vancomycin and caspofungin therapy by 24 hours. Our findings suggest that current empirical treatment strategies predominantly target Gram-negative bacteremia, potentially overlooking initial coverage for Gram-positive and fungal BSIs. In addition, antimicrobial resistance was associated with delays, ddPCR testing may be a key for earlier adequate antimicrobial treatment for MDRO infections (Enne et al., 2025). However, ddPCR-guided therapy was associated with significantly lower in-hospital mortality, particularly in high-risk subgroups (Gram-negative, MDRO, and persistent BSI), highlighting its clinical value for targeted antimicrobial management. Our patient cohort, marked by high mortality, complicated intra-abdominal infections, and approximately 30% prevalence of drug-resistant organisms, underscores the essential clinical utility of ddPCR in this setting. Moreover, determining the optimal treatment duration and identifying significant outcomes for study remains challenging. Large, multicenter trials should be conducted to verify the role of ddPCR in directing antimicrobial treatment for various infections, including respiratory and urinary tract infections.

More critically, given the rapid clinical deterioration often observed in sepsis patients, quantitative assessment of microbial burden serves as the most direct indicator for evaluating antimicrobial treatment efficacy, particularly when integrated with therapeutic drug monitoring (Abasıyanık et al., 2020). Emerging evidence from quantitative molecular studies, particularly real-time PCR-based analyses, supports this observation. In a study of 51 critically ill patients with *Acinetobacter baumannii* bacteremia, where serial real-time PCR measurements of bacterial DNA load served as a sensitive biomarker for monitoring antibiotic treatment response (Chuang et al., 2010). Previous quantitative results from ddPCR demonstrated strong concordance with both CRP and PCT levels, these findings support the utility of ddPCR for dynamic monitoring of infection severity and for guiding antibiotic stewardship decisions (Li et al., 2025). Our findings show that ddPCR-guided therapy resulted in a significantly greater reduction in CRP levels compared to standard care, which was associated with improved clinical outcomes. However, clinicians should interpret positive results with caution, as they may reflect either false positives or colonization rather than active infection. Additionally, since molecular methods cannot distinguish between viable and non-viable organisms, this limitation may result in underestimated pathogen clearance rates. The prolonged positivity of ddPCR may extend antimicrobial therapy duration and affect de-escalation decisions, warranting further research on its role in treatment monitoring and guiding antibiotic discontinuation. Current blood culture evidence supports short-course therapy (The BALANCE Investigators, 2025). Clinicians should assess symptomatic improvement and integrate dynamic changes in biomarkers and molecular diagnostics to guide antibiotic cessation. Furthermore, in hemodynamically unstable patients, caution should be exercised when considering early discontinuation of broad-spectrum antibiotics based on negative ddPCR results, as this may miss pathogens not covered by the panel and lead to adverse outcomes (Enne et al., 2025). Future therapeutic strategies should incorporate dynamic monitoring of PCT, CRP, and IL-6 to guide antibiotic stewardship.

Our study has several limitations. First, it is an observational, single-center investigation with a relatively small sample size. Although a difference in the primary endpoint was observed, it did not reach statistical significance. Second, the lack of randomization may have introduced selection bias. Additionally, the non-blinded design could have led to subjective influences on both the testing process and antibiotic treatment decisions. However, statistical methods including multivariate regression and IPTW were employed to adjust for potential confounding factors.

## Conclusion

In summary, integrating ddPCR into routine ICU antimicrobial stewardship shortened time to optimal therapy and conferred survival benefits in selected BSI subgroups. As a powerful complement to conventional blood cultures, ddPCR achieves maximal benefit when combined with serial biomarker monitoring and expert clinical interpretation.

## Data Availability

The raw data supporting the conclusions of this article will be made available by the authors, without undue reservation.
