# Supplementary material for: Impact of ddPCR panel implementation on optimizing antimicrobial therapy management in critically ill patients with bloodstream infections
Source: Front Cell Infect Microbiol. 2026 Jul 6;16:1850336. doi: 10.3389/fcimb.2026.1850336 (PMC13381250; doi:10.3389/fcimb.2026.1850336)
Supplement: Supplementary file 1 [file Table1.docx]

**Title Page**

**Impact of ddPCR panel implementation on optimizing antimicrobial therapy management in critically ill patients with bloodstream infections**

Xiaoli Wang ^1†^,Jing Wu ^1†^,Chengcheng Shen ^1†^,Rui Tian ^1^,Yunqi Dai ^1^,Jingjing Huang ^2^,De-en Zhong ^1^,Danting Fang ^1^,Jingyong Sun ^3^, Hongping Qu^1,4∗^,Ruoming Tan ^1,4∗^

^1^Department of Critical Care Medicine, Ruijin Hospital, Shanghai Jiao Tong University School of Medicine, Shanghai 200025, China

^2^Department of Pharmacy, Ruijin Hospital, Shanghai Jiao Tong University School of Medicine, Shanghai 200025, China

^3^Department of Clinical Microbiology, Ruijin Hospital, Shanghai Jiao Tong University School of Medicine, Shanghai 200025, China

^4^College of Health Science and Technology, Shanghai Jiao Tong University School of Medicine, Shanghai 200025, China

^†^ Xiaoli Wang, Jing Wu and Chengcheng Shen contributed equally to this work.

^∗^Corresponding author. Author order was determined by work allocation.

Correspondence and requests for materials should be addressed to:

Hongping Qu ([hongpingqu0412@hotmail.com](mailto:hongpingqu0412@hotmail.com));

Ruoming Tan ([sandratan37@hotmail.com](mailto:sandratan37@hotmail.com))

**Table S1.** **Univariate and multivariate** **Poisson Regression between the ddPCR-guided group and standard-of-care group.**

| **Clinical characteristics** | **Univariate Poisson Regression** | | | **Multivariate Poisson Regression** | | |
| --- | --- | --- | --- | --- | --- | --- |
|  | **IRR** | **95% CI** | **p-value** | **IRR** | **95% CI** | **p-value** |
| Standard-of-care group | 1.52 | (0.92, 2.55) | 0.10 | 1.59 | (0.95, 2.72) | 0.082 |
| Gender: Male | 0.83 | (0.51, 1.39) | 0.48 |  |  |  |
| Age | 1.00 | (0.99, 1.02) | 0.61 |  |  |  |
| **Comorbidities** |  |  |  |  |  |  |
| Hypertension | 0.97 | (0.59, 1.61) | 0.89 |  |  |  |
| Diabetes mellitus | 1.32 | (0.77, 2.19) | 0.31 |  |  |  |
| Coronary heart disease | 0.97 | (0.49, 1.75) | 0.92 |  |  |  |
| Chronic obstructive pulmonary disease | 0.73 | (0.04, 3.28) | 0.74 |  |  |  |
| Chronic kidney disease | 1.73 | (0.86, 3.19) | 0.12 |  |  |  |
| Malignant tumor | 0.83 | (0.50, 1.37) | 0.47 |  |  |  |
| Immunosuppressive | 1.11 | (0.53, 2.09) | 0.76 |  |  |  |
| **Main source of targeted organisms** |  |  | 0.52 |  |  |  |
| Abdominal | - | - |  |  |  |  |
| Respiratory | 1.20 | (0.72, 2.00) |  |  |  |  |
| Urine | 0.00 | (0.00, Inf) |  |  |  |  |
| Skin and soft | 0.00 | (0.00, Inf) |  |  |  |  |
| Catheter-related | 1.16 | (0.36, 3.80) |  |  |  |  |
| Others | 0.00 | (0.00, Inf) |  |  |  |  |
| **Identified pathogen** |  |  |  |  |  |  |
| Gram-positive organism | 0.95 | (0.54, 1.61) | 0.86 |  |  |  |
| Gram-negative organism | 1.14 | (0.69, 1.88) | 0.61 |  |  |  |
| Fungus | 1.07 | (0.57, 1.89) | 0.82 |  |  |  |
| Receive optimal antibiotic therapy on enrollment | 1.10 | (0.63, 1.85) | 0.74 |  |  |  |
| Mechanical ventilation | 2.42 | (1.13, 6.27) | 0.021 | 2.37 | (1.08, 6.25) | 0.050 |
| Septic shock | 1.80 | (0.96, 3.76) | 0.069 | 1.22 | (0.64,2.60) | 0.6 |
| APACHE II score | 1.01 | (0.99, 1.04) | 0.36 |  |  |  |
| SOFA score | 1.04 | (0.99, 1.09) | 0.13 |  |  |  |
| Surgery performed before 30 days of inclusion | 1.08 | (0.65, 1.81) | 0.77 |  |  |  |
| **Clinical indicators** |  |  |  |  |  |  |
| C Reactive Protein, mg/L, median (IQR) | 1.00 | (1.00, 1.00) | 0.33 |  |  |  |
| White Blood Cell,10^9/L, median (IQR) | 1.01 | (0.97, 1.04) | 0.66 |  |  |  |
| Lymphocyte, 10^9/L, median (IQR) | 0.95 | (0.65, 1.17) | 0.69 |  |  |  |
| Platelet, 10^9/L, median (IQR) | 1.0 | (0.99, 1.00) | <0.001 | 1.00 | (0.99, 1.00) | 0.021 |
| Procalcitonin, ng/mL, median (IQR) | 1.00 | (1.00, 1.01) | 0.20 |  |  |  |
| Pro-BNP, ng/mL, median (IQR) | 1.00 | (1.00, 1.00) | 0.042 | 1.00 | (1.00, 1.00) | >0.9 |
| Pre-albumin, g/L, median (IQR) | 0.99 | (0.99, 1.00) | 0.023 | 0.99 | (0.99, 1.00) | 0.070 |
| Creatinine, μmol/L, median (IQR) | 1.00 | (1.00, 1.00) | 0.037 | 1.00 | (1.00, 1.00) | 0.2 |
| Total Bilirubin, μmol/L, median (IQR) | 1.00 | (1.00, 1.00) | 0.071 | 1.00 | (1.00, 1.00) | 0.4 |
| Fibrinogen, g/L, median (IQR) | 1.00 | (0.96, 1.03) | 0.83 |  |  |  |
| D-dimer, μg/L, median (IQR) | 1.01 | (0.97, 1.04) | 0.72 |  |  |  |

Notes:

IRR, Incidence Rate Ratio; CI, Confidence Interval.

**Table S2. Changes in clinical indicators from enrollment to Day 5 in the ddPCR-guided group and standard-of-care group.**

| **Clinical characteristics** | **ddPCR-guided**  **group (n=69)** | **Standard-of-care**  **group(n=69)** | **P value** |
| --- | --- | --- | --- |
| C Reactive Protein, mg/L, median (IQR) | -49(-115, -11) | -7(-50,29) | 0.009* |
| White Blood Cell,10^9/L, median (IQR) | 0.5(-3.3,3.2) | -1.6(-4.8,1.4) | 0.114 |
| Lymphocyte, 10^9/L, median (IQR) | 0.04(-0.08,0.3) | 0.04(-0.18,0.34) | 0.314 |
| Platelet, 10^9/L, median (IQR) | 8(-44,71) | 0(-34,43) | 0.842 |
| Procalcitonin,ng/mL, median (IQR) | -0.8(--4.6,-0.0) | -0.5(-5.5,0.3) | 0.382 |
| Pro-BNP, ng/mL, median (IQR) | 152(-1049,1960) | -334(-1321,280) | 0.997 |
| Pre-albumin, g/L, median (IQR) | 10(-31,39) | -1.5(-24,22) | 0.386 |
| Creatinine, μmol/L, median (IQR) | -6(-25,12) | -8(-50,13) | 0.174 |
| Total Bilirubin, μmol/L, median (IQR) | -1.0(-7.0,8.7) | -0.6(-7,8.7) | 0.971 |
| Fibrinogen, g/L, median (IQR) | -0.2(-1.2,0.4) | -0.4(-1.6,0.6) | 0.615 |
| D-dimer, μg/L, median (IQR) | 0.1(-1.4,2.1) | -0.6(-3.1,1.1) | 0.266 |

**Fig.S1. Flowchat of the study**

**
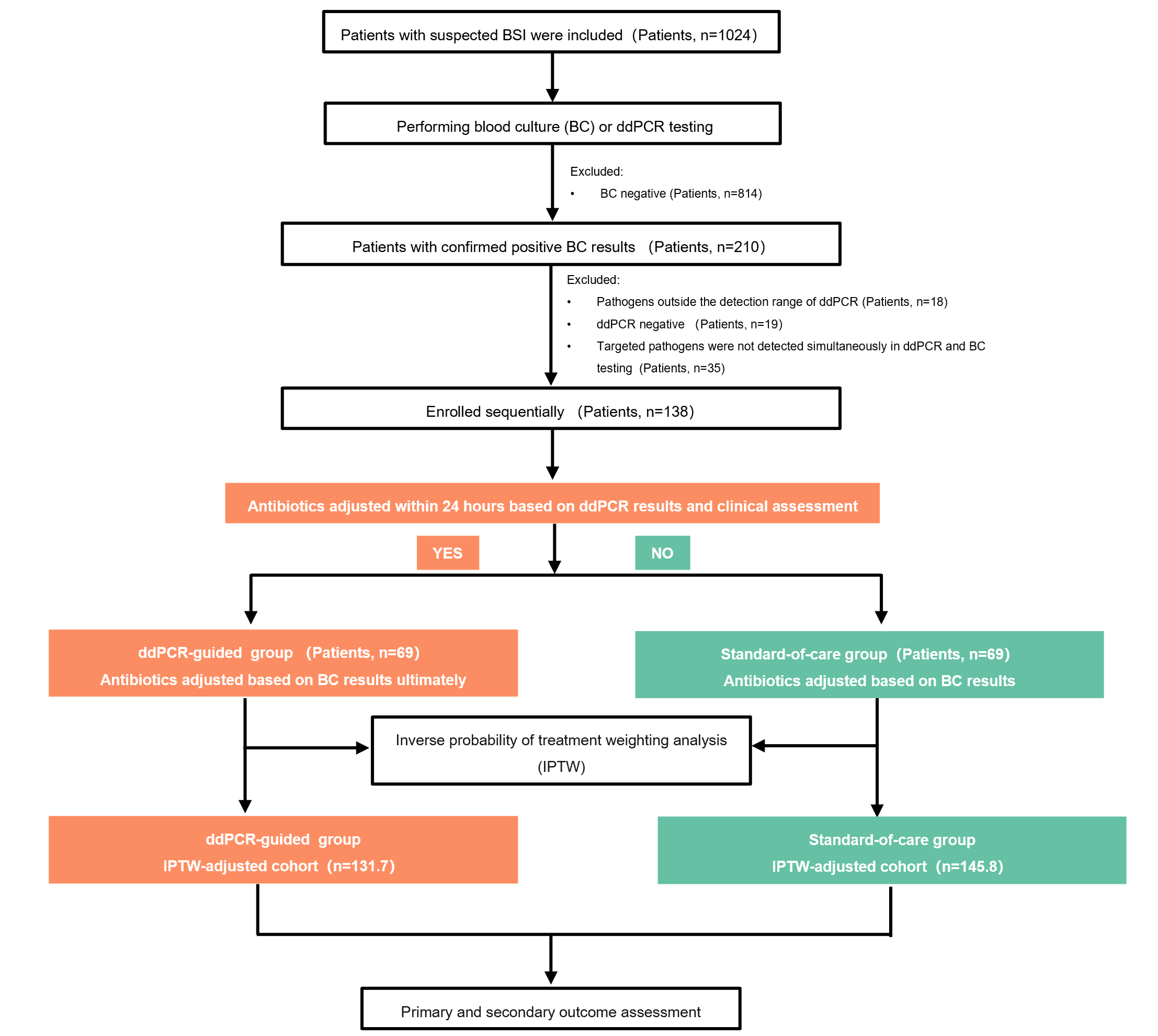
**

**Fig.S2. Proportion of missing values for variables with missing data.**

**
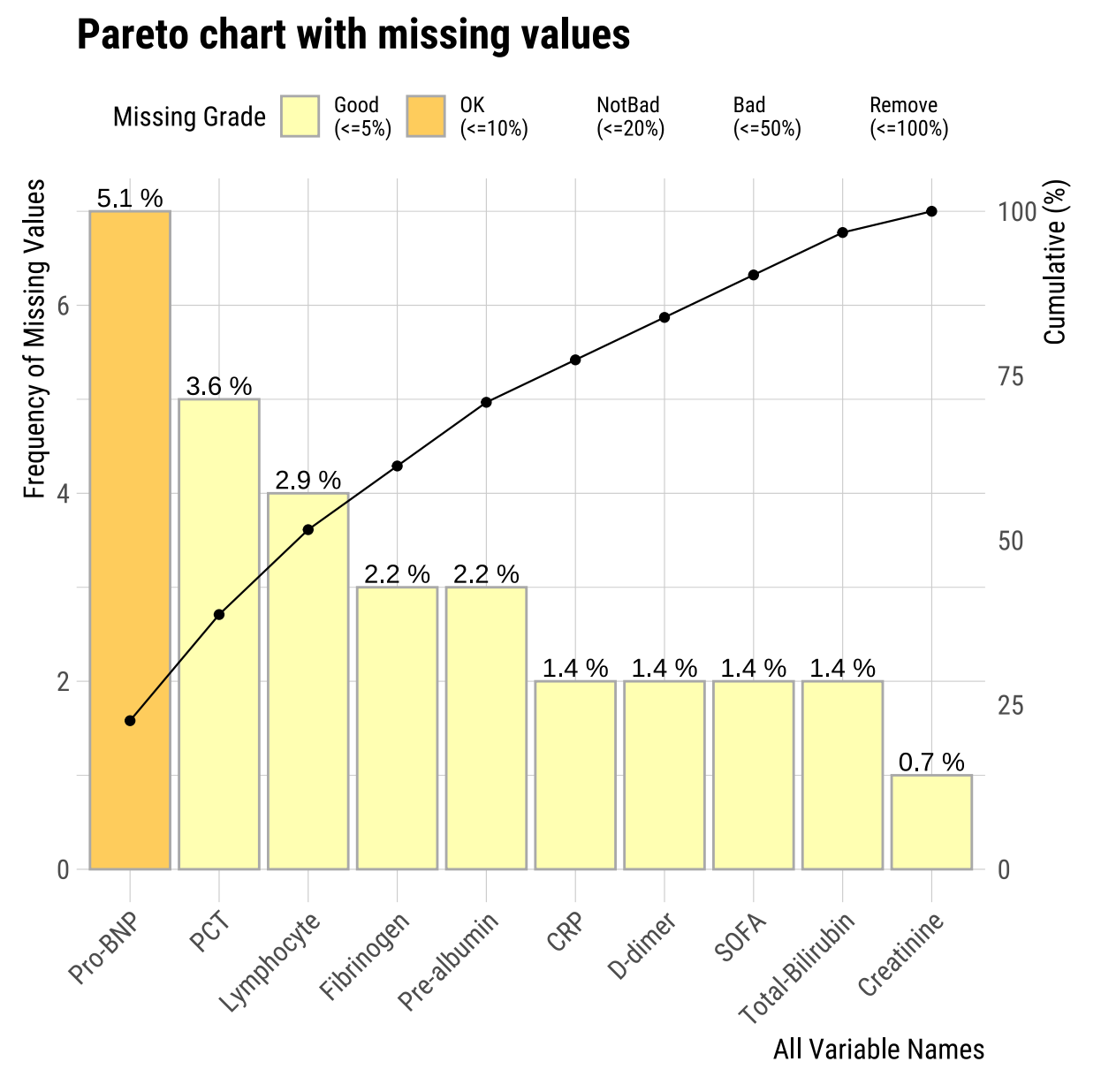
**

The bar plot displays the percentage of missing data for each variable among all patient.

**Notes**

**Supplementary Data**

The datasets used and/or analyzed in the current study are available from the corresponding author on reasonable request.

**List of abbreviations**

BSIs: bloodstream infections; ICU: intensive care unit; BCs: blood cultures; ddPCR: droplet digital PCR; AMR: antimicrobial resistance; PCT: procalcitonin; IL-6: interleukin-6; AST: antimicrobial susceptibility testing; MDROs: multidrug-resistant organisms; CRP: C-reactive protein; SSC: Surviving Sepsis Campaign; mPCR: multiplex PCR; IPTW: inverse probability of treatment weighting; SMD: standardized mean difference; IRR: incidence rate ratio.
